# Supplementary material for: Bioactive Phenolic Compounds from Rambutan (Nephelium lappaceum L.) Shell: Encapsulation, Structural Stability, and Multifunctional Activities
Source: Int J Mol Sci. 2025 Nov 9;26(22):10859. doi: 10.3390/ijms262210859 (PMC12652491; doi:10.3390/ijms262210859)
Supplement: Supplementary file 1 [file ijms-26-10859-s001.zip › ijms-3891764-supplementary.pdf]

Supplementary material

Bioactive Phenolic Compounds from Rambutan (*Nephelium lappaceum* L.) Shell: Encapsulation, Structural Stability, and Multifunctional Activities

Carlos Barba-Ostria [1,2,*], Orestes López [3], Alexis Debut [4,5], Arianna Mayorga-Ramos [6], Johana Zúñiga-Miranda [6], Elena Coyago-Cruz [7], Rebeca Gonzalez-Pastor [6], Kevin Cartuchi [3], Antonella Viteri [6], Ana Belén Peñaherrera-Pazmiño [6] and Linda P. Guamán [6,*]

1 Escuela de Medicina, Colegio de Ciencias de la Salud Quito, Universidad San Francisco de Quito (USFQ), Quito 170901, Ecuador
2 Instituto de Microbiología, Universidad San Francisco de Quito (USFQ), Quito 170901, Ecuador
3 Facultad de Ciencia e Ingeniería en Alimentos y Biotecnología, Universidad Técnica de Ambato, Ambato 180104, Ecuador; od.lopez@uta.edu.ec (O.L.); ramirocartuchi@gmail.com (K.C.)
4 Centro de Nanociencia y Nanotecnología, Universidad de Las Fuerzas Armadas ESPE, Sangolquí 171103, Ecuador; apdebut@espe.edu.ec
5 Departamento de Ciencias de la Vida y Agricultura, Universidad de las Fuerzas Armadas ESPE, Sangolquí 171103, Ecuador
6 Centro de Investigación Biomédica (CENBIO), Facultad de Ciencias de la Salud Eugenio Espejo, Universidad UTE, Quito 170527, Ecuador; arianna.mayorga@ute.edu.ec (A.M.-R.); johana.zuniga@ute.edu.ec (J.Z.-M.); rebeca.gonzalez@ute.edu.ec (R.G.-P.); antonellaviteri28@gmail.com (A.V.); ana.penaherrera@ute.edu.ec (A.B.P.-P.)
7 Carrera de Ingeniería en Biotecnología de los Recursos Naturales, Universidad Politécnica Salesiana, Sede Quito, Campus El Girón, Av. 12 de Octubre N2422 y Wilson, Quito 170143, Ecuador; ecoyagoc@ups.edu.ec
* Correspondence: cbarbao@usfq.edu.ec (C.B.-O.); linda.guaman@ute.edu.ec (L.P.G.); Tel.: +593-2-2990800 (ext. 2115) (C.B.-O. & L.P.G.)

Figure S1. Scavenging activity of microencapsulated *N. lappaceum*

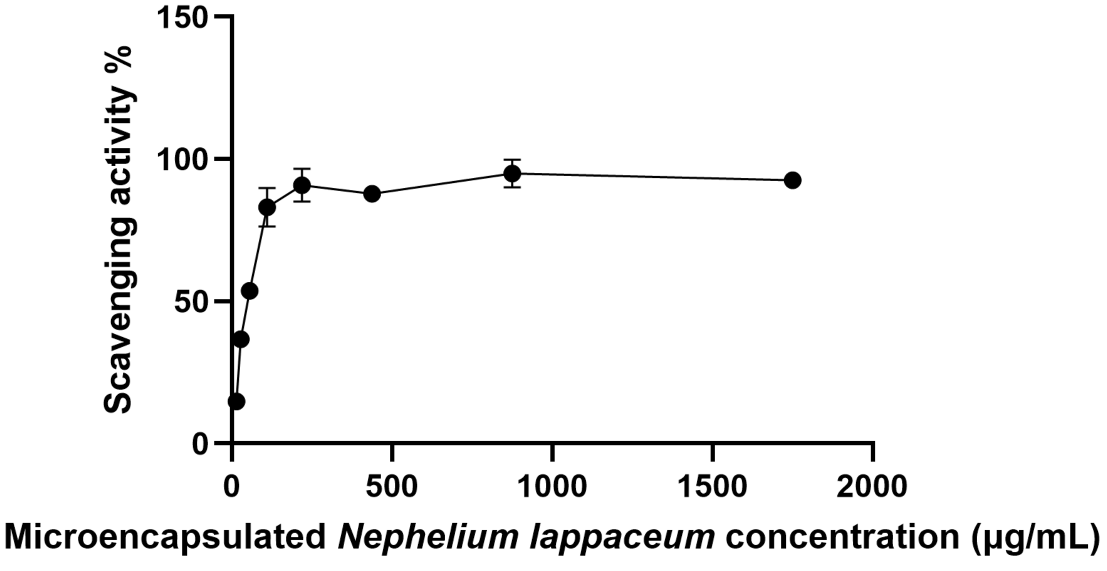

Table S1. Antifungal activity of microencapsulated *Nephelium lappaceum* shell extract against *Candida albicans*, *C. glabrata*, *C. krusei*, and *C. tropicalis*.

**Condition**	**Final compound concentration (mg mL^{-1})**	***C. albicans* growth**	***C. glabrata* growth**	***C. krusei* growth**	***C. tropicalis* growth**
Treated	125	Lawn growth	Lawn growth	Lawn growth	Lawn growth
Control (YPD + water)	0	Lawn growth	Lawn growth	Lawn growth	Lawn growth
Control (YPD + compound, no cells)	125	N/A	N/A	N/A	N/A

Table S2. Evaluation of Biofilm Inhibition activity (microencapsulated *N. lappaceum* extract)

**Micro**	***Staphylococcus aureus*** **ATCC 25923**					***Listeria monocytogenes*** **ATCC 13932**					***Pseudomonas aeruginosa*** **ATCC 9027**					***Burkholderia cepacia*** **ATCC 25416**				
**ug/mL**	**Inh percentage**			**Mean**	**SD**	**Inh percentage**			**Mean**	**SD**	**Inh percentage**			**Mean**	**SD**	**Inh percentage**			**Mean**	**SD**
**10**	84%	87%	86%	86%	1,5%	96%	99%	99%	98%	1,7%	N-Inh	N-Inh	N-Inh	-	-	68%	45%	63%	59%	12,4%
**5**	86%	87%	85%	86%	1,1%	94%	96%	95%	95%	1,0%	N-Inh	N-Inh	N-Inh	-	-	39%	19%	21%	26%	11,0%
**1**	86%	88%	86%	87%	1,0%	94%	94%	92%	93%	1,4%	N-Inh	N-Inh	N-Inh	-	-	N-Inh	N-Inh	N-Inh	-	-
**0,1**	79%	84%	80%	81%	2,9%	59%	75%	67%	67%	8,1%	9%	5%	4%	6%	2,9%	N-Inh	N-Inh	N-Inh	-	-
**0,01**	13%	20%	4%	13%	8,3%	N-Inh	N-Inh	N-Inh	-	-	33%	19%	29%	27%	7,1%	N-Inh	N-Inh	N-Inh	-	-
**0,001**	N-Inh	N-Inh	N-Inh	-	-	N-Inh	N-Inh	N-Inh	-	-	26%	9%	28%	21%	10,5%	N-Inh	N-Inh	N-Inh	-	-

***N-Inh:** No inhibition of microorganism biofilm growth at the described concentration
